# Spatial differences influence nitrogen uptake, grain yield, and land-use advantage of wheat/soybean relay intercropping systems

**DOI:** 10.1038/s41598-023-43288-3

**Published:** 2023-10-07

**Authors:** Muhammad Ali Raza, Atta Mohi Ud Din, Wang Zhiqi, Hina Gul, Sana Ur Rehman, Birra Bukhari, Imran Haider, Muhammad Habib Ur Rahman, Xue Liang, Shuanglong Luo, Ayman El Sabagh, Ruijun Qin, Ma Zhongming

**Affiliations:** 1 Gansu Academy of Agricultural Sciences, Lanzhou, China; 2 National Research Center of Intercropping, The Islamia University of Bahawalpur, Bahawalpur, Pakistan; 3 National Center for Industrial Biotechnology, Arid Agricultural University, Rawalpindi, Pakistan; 4 College of Life Sciences, South China Agricultural University, Guangzhou, China; 5 Department of Seed Science and Technology, MNS University of Agriculture, Multan, Pakistan; 6 Department of Field Crops, Faculty of Agriculture, Siirt University, Siirt, Turkey; 7 Hermiston Agricultural Research and Extension Center, Oregon State University, Corvallis, USA

**Keywords:** Ecology, Plant sciences, Ecology, Environmental sciences

## Abstract

Cereal/legume intercropping is becoming a popular production strategy for higher crop yields and net profits with reduced inputs and environmental impact. However, the effects of different spatial arrangements on the growth, grain yield, nitrogen uptake, and land-use advantage of wheat/soybean relay intercropping are still unclear, particularly under arid irrigated conditions. Therefore, in a three-year field study from 2018 to 2021, soybean was relay intercropped with wheat in different crop configurations (0.9 m, narrow strips; 1.8 m, medium strips; and 2.7 m, wide strips), and the results of intercropping systems were compared with their sole systems. Results revealed that intercrops with wide strips outperformed the narrow and medium strips, when the objective was to obtain higher total leaf area, dry matter, nitrogen uptake, and grain yield on a given land area due to reduced interspecific competition between intercrops. Specifically, at maturity, wide strips increased the dry matter accumulation (37% and 58%) and its distribution in roots (37% and 55%), straw (40% and 61%), and grains (30% and 46%) of wheat and soybean, respectively, compared to narrow strips. This enhanced dry matter in wide strips improved the soybean’s competitive ability (by 17%) but reduced the wheat’s competitive ability (by 12%) compared with narrow strips. Noticeably, all intercropping systems accumulated a significantly higher amount of nitrogen than sole systems, revealing that wheat/soybean relay intercropping requires fewer anthropogenic inputs (nitrogen) and exerts less pressure on the ecosystem than sole systems. Overall, in wide strips, intercropped wheat and soybean achieved 62% and 71% of sole wheat and soybean yield, respectively, which increased the greater total system yield (by 19%), total land equivalent ratio (by 24%), and net profit (by 34%) of wide strips compared to narrow strips. Our study, therefore, implies that the growth parameters, grain yields, nutrient accumulation, and land-use advantage of intercrop species could be improved with the proper spatial arrangement in cereal/legume intercropping systems.

## Introduction

Intercropping systems are practiced widely all over the globe, especially in developing countries like China, India, and Pakistan^1^. These systems are environmentally friendly as they reduce the nitrogen input and nitrate-nitrogen accumulation in soil profiles^2^. Notably, intercropping is sustainable and recommended to be utilized on a large scale^3^, not only in irrigated areas^4^ but also in rainfed areas^5^ because it produces a higher total grain yield than the grain yield of the sole system^6^. For example, wheat and soybean produced greater yields in wheat/soybean intercropping systems than in sole systems^7^. The intercropping of soybean with wheat achieved a 30% higher land-use advantage than their sole systems^8^. The stable and higher grain yield of component crops in intercropping systems is mainly attributed to resource complementarity, in which component crops utilize land, water, and light more efficiently and effectively due to different temporal^9^, spatial^10^, crop growth stages^11^, and cogrowth period^12^. However, among various factors, the selection of crop combinations in intercropping determines the positive or negative resource complementarity^1,3^. Thus, selecting appropriate crop combinations, e.g., cereals with legumes (soybean), particularly with reduced inputs (nutrients), is critical for the productivity and large-scale adoption of intercropping systems.

Recent research has stressed the inclusion of temporal dynamics (sowing of crops at different times) in competition and nutrient uptake studies^13,14^. Most of the previous work examines the dynamics of intercrops based on the temporal segregation of biomass accumulation or yield and yield components. For example, Dong et al. (2018) observed temporal niche complementarity between intercropped species due to differences in sowing and harvesting dates of intercrops in oilseed rape/soybean relay intercropping system ^15^; Gou et al.^16^ obtained maximum land-use advantage in wheat/maize relay intercropping, where maize was planted 54 days after wheat sowing. Similarly, Wu et al.^17^ reported temporal niche differentiation for biomass accumulation between maize and soybean in maize/soybean relay intercropping, where maize was sown ∼60 days before soybean sowing. Though soybean growth was negatively affected during the cogrowth phase with maize, but they observed a temporal shift in acquiring the available resources, and soybean showed strong recovery growth after the harvest of maize in maize soybean relay intercropping. On the other hand, some studies have reported a positive coexistence of intercrops^18,19^, which reflects the phenological shifts in using the available resources, which promote complementary and facilitation interactions and suppress competitive interactions between intercrops. Importantly, in relay intercropping systems, intercrop captures resources at different times and avoids competition with neighboring crop species^9,20^. These responses are important in increasing the total system yield of intercropping systems by improving niche complementarity^21–24^. However, studies focusing on temporal dynamics of nutrient uptake, particularly of nitrogen uptake and distribution in different plant parts of intercrops under arid-irrigated conditions, are rare. Therefore, determining the effects of the temporal difference on nitrogen uptake and distribution in intercrops is extremely valuable for tracking the reasons for high yield and land-use advantage in relay intercropping systems.

Almost all the main intercropping systems, maize/soybean^3^, maize/potato^25^, maize/peanut^26^, wheat/maize^27^, and wheat/chickpea^8^, are used with a low level of mechanization^28,29^. In addition, the low labor income from farming activities and the shift of rural labor into other sectors exert extra pressure to produce higher crop yields on these systems^30^. However, intercropping systems with a higher level of mechanization have significant potential to enhance the land productivity and net income of farmers^28^, which ultimately can also solve the problem of rural labor scarcity for field operations^4^. For mechanization, the conventional intercropping needs to be modified into strip intercropping^1^, and wide strips could be used for intercropping systems that could be mechanized using small farm machinery without losing the synergistic effects between intercrops^25^. Typically, in strip intercropping, farmers plant two crops in separate strips on the same land for a specific growth period^31^, which allows positive interspecific interactions^32^, facilitation in land and light utilization^33^, water uptake^34^, and nutrient accumulation^18^. The degree of these mutualistic interactions directly correlates with the strip distance between intercrops^10,33,35^. Several scientists evaluated the performance of strip intercropping systems with wide strips; they found that strip intercropping systems with a wide strip of 8 m^36^, 6 m^37^, 3.3 m^31^, 3.1 m^38^, 3 m^39^, and 2.4 m^4^ achieved the higher values of land equivalent ratio (LER) than narrow strips. However, the use of wide strips in intercropping systems is also criticized by some researchers^40,41^, and they obtained maximum benefits from intercropping with narrow strips of one or two m^42,43^. These findings reinforce the abovementioned concerns that farmers will not adopt those intercropping systems, which restricts the use of farm machinery, especially in the presence of mechanized large-scale sole systems^28,30^.

Cereal/legume combinations in relay intercropping systems are generally very effective due to complementary nitrogen use strategies. The cereals, e.g., wheat and maize, are the dominant crop species in nitrogen uptake and use in intercropping, which forces the neighboring legumes, e.g., soybean, peanut, and chickpea, to fix atmospheric nitrogen^44^, resulting in complementary nitrogen use in intercropping. Hence, facilitation and complementary processes are largely involved in enhancing the land use advantage of cereal/legume intercropping systems. However, how spatial differences influence the resource (land and nitrogen) acquisition between wheat and soybean in wheat/soybean relay intercropping systems are rarely investigated. Therefore, in this large field experiment for three years, the effects of different strip widths were evaluated on the performance of intercrops in wheat/soybean relay intercropping systems (WSI). Thus, we hypothesize that (i) wheat and soybean will produce greater dry matter and grain yield with wide strips than narrow strips in WSI; (ii) due to the temporal differences, intercropping systems will uptake higher total nitrogen (wheat nitrogen uptake + soybean nitrogen uptake) than sole wheat or soybean, and (iii) the wide operating strips will be easier to manage, and it will generate more net profit than narrow strips in WSI.

## Results

### Leaf area index (LAI) of wheat and soybean

**Figure 1:**
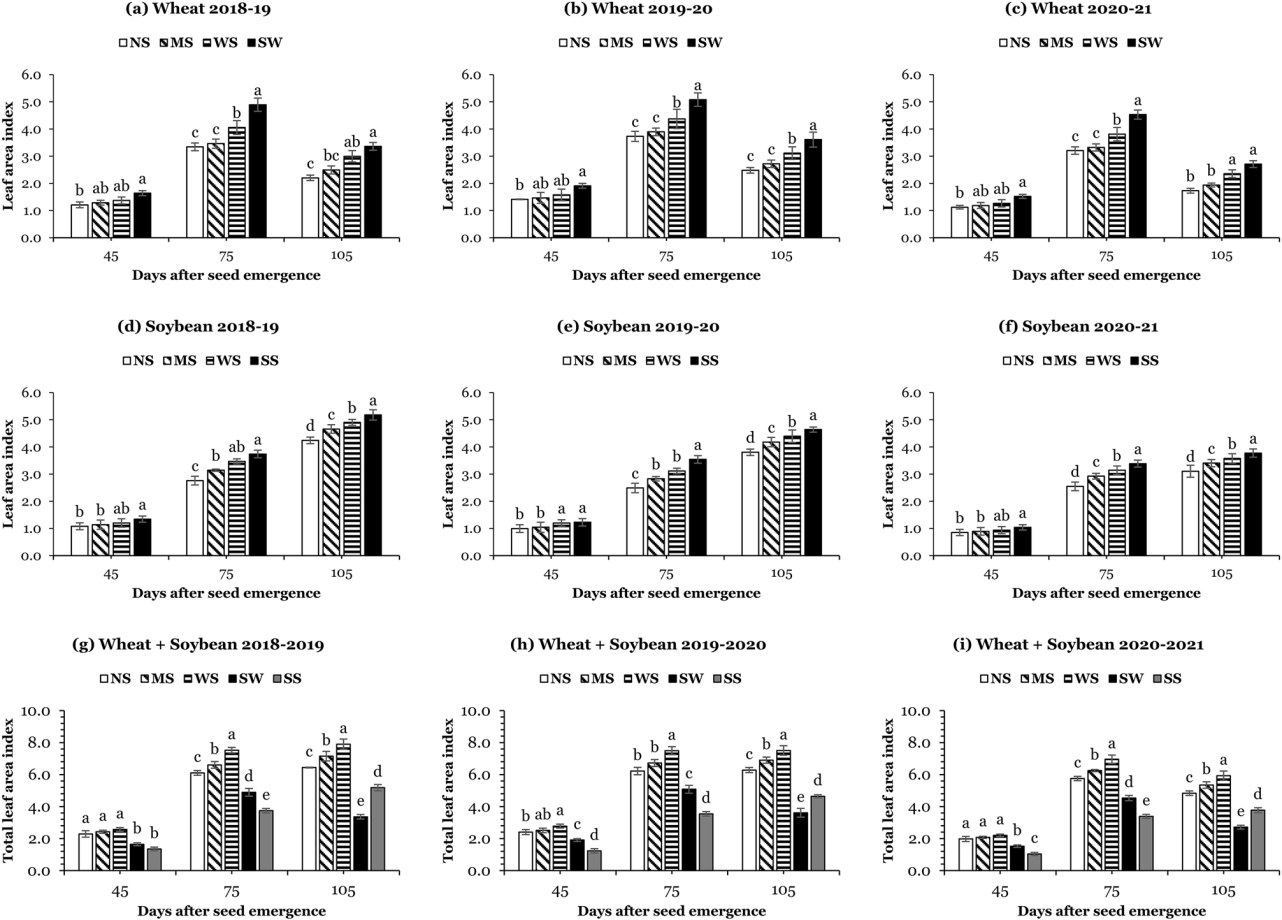
Leaf area index of wheat (**a**–**c**) and soybean (**d**–**f**), and total leaf area index (**g**–**i**) at 45, 75, and 105 days after seed emergence under different planting systems: SW (sole wheat), SS (sole soybean), NS (narrow strips; 0.9 m), MS (medium strips; 1.8 m), and WS (wide strips; 2.7 m). Means are averages over three replicates ± standard error of the mean.

Leaf area dynamics over time changed for both crops. In this study, the maximum LAI for wheat was noticed at 75 days after seed emergence (DAS), while for soybean, it was observed at 105 DAS. In WSI, the first-sown wheat covered the land at the start of the season, and the second-sown soybean covered the land at the end of the season. On average, over the years, intercropped wheat + soybean covered the ground for an extra 66 ± 02 days in WSI than the growing season of sole wheat or sole soybean. At all sampling stages and years, different planting systems (sole and strip intercropping) significantly influenced the LAI of wheat (Fig. 1a–c) and soybean (Fig. 1d–f). At 45, 75, and 105 DAS, the mean maximum LAI of both crops was observed in sole systems, while the mean minimum LAI of both crops was noticed in narrow strips. In WSI, at 105 DAS, the average highest LAI of wheat (3.2) and soybean (4.5) was recorded in wide strips, whereas the average lowest LAI of wheat (2.1) and soybean (3.7) was noted in narrow strips. However, the total LAI of intercropping systems was significantly higher than the LAI of sole wheat and sole soybean (Fig. 1g–i). For instance, at 105 DAS, the total LAI in narrow strips, medium strips, and wide strips, respectively, was increased by 81%, 100%, and 120% compared to sole wheat and by 29%, 43%, and 57% compared to sole soybean, indicating that the WSI as a whole developed better canopy to capture solar radiations.

### Dry matter accumulation and distribution in wheat and soybean

**Figure 2:**
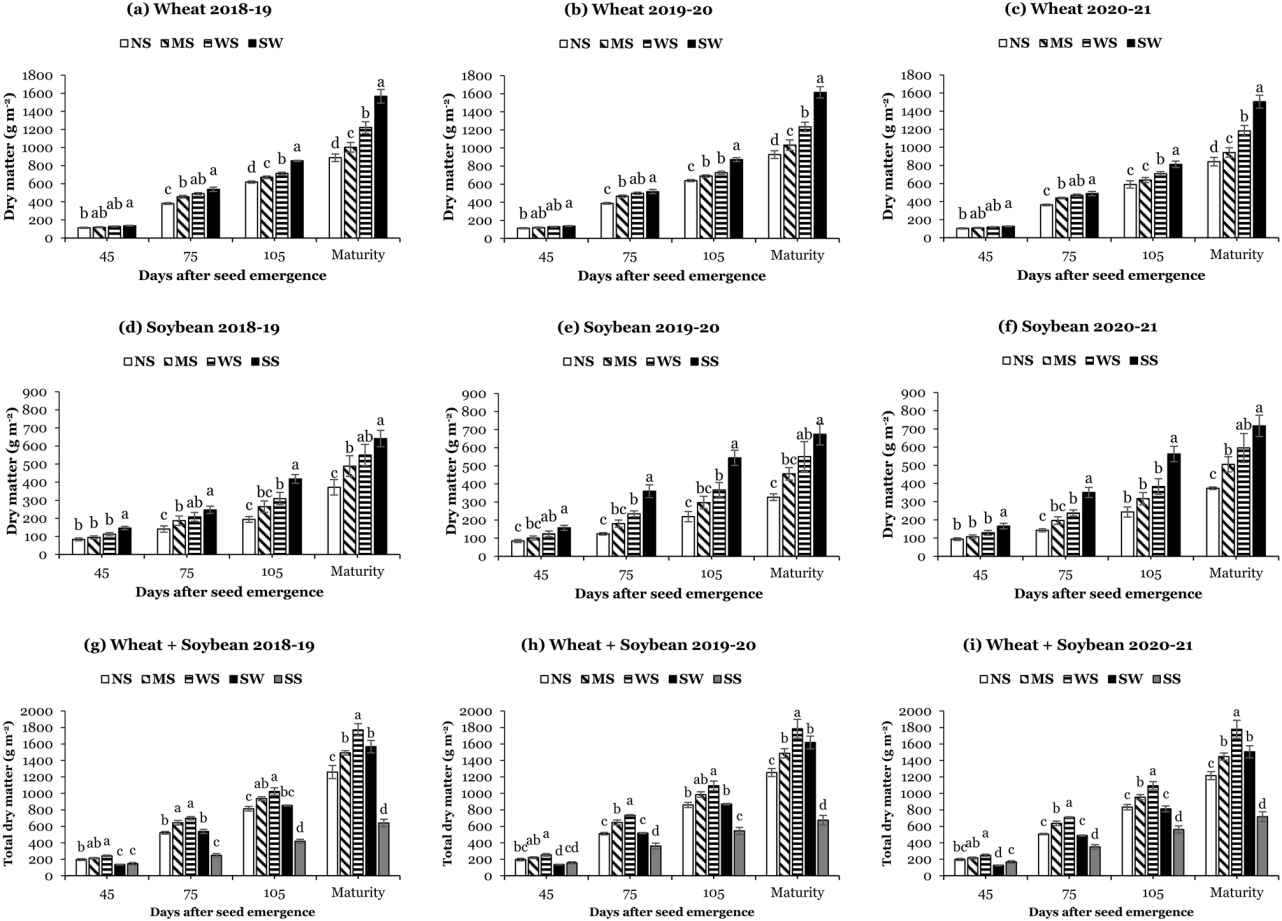
Dry matter of wheat (**a**–**c**) and soybean (**d**–**f**), and total dry matter (**g**–**i**) at 45, 75, and 105 days after seed emergence and maturity under different planting systems: SW (sole wheat), SS (sole soybean), NS (narrow strips; 0.9 m), MS (medium strips; 1.8 m), and WS (wide strips; 2.7 m). Means are averages over three replicates ± standard error of the mean.

The different planting systems significantly affected the dry matter of wheat (Fig. 2a–c) and soybean (Fig. 2d–f). The dry matter of both crops in all systems increased rapidly from 45 to 105 DAS and achieved the highest values at maturity. Average over the years, at maturity, the maximum (1562.4 g m^−2^) dry matter of wheat in sole wheat was 77%, 57%, and 29% higher than the dry matter of wheat in narrow strips, medium strips, and wide strips, respectively; whereas, the maximum (677.6 g m^−2^) soybean dry matter in sole soybean was 90%, 40%, and 20%, higher than the dry matter of soybean in narrow strips, medium strips, and wide strips, respectively. In contrast, at all sampling times, the total dry matter of WSI was significantly higher than the dry matter of sole systems (Fig. 2g–i), and the total dry matter of different planting systems exhibited the trend as wide strips > sole wheat > medium strips > narrow strips > sole soybean. Overall, among WSI, at maturity, the total dry matter in wide strips was increased by 43% and 21% compared to narrow and medium strips, respectively. Besides, the different planting systems significantly changed the dry matter distribution in plant organs of wheat (Table 1) and soybean (Table 2) at 45, 75, and 105 DAS and maturity, except at the first sampling stage (45 DAS) of wheat. Across the years, at maturity, wheat distributed the highest dry matter in the roots (189.9 g m^−2^), straw (962.9 g m^-2^), and grains (409.6 g m^−2^) in sole wheat, while the highest dry matter in the roots (101.5 g m^−2^), straw (476.9 g m^−2^), and grains (99.2 g m^−2^) of soybean were noticed in sole soybean. However, in WSI, compared to narrow strips, wide strips increased the distribution of dry matter in the grains of wheat and soybean by 30% and 46%, respectively. Additionally, in all years of this study, the dynamics of dry matter accumulation and its distribution at maturity under different planting systems were consistent with those of the other sampling times (75 and 105 DAS).

**Table 1:** Dry matter distribution in wheat at 45, 75, and 105 days after seed emergence (DAS) and maturity under different planting systems.

Years	Planting systems	Wheat dry matter distribution (g m^-2^)								
		45 DAS		75 DAS		105 DAS		Maturity		
		Root	Straw	Root	Straw	Root	Straw	Root	Straw	Grains
2018–19	Narrow strips	12.0 ± 1.8^NS^	100.8 ± 8.0^NS^	40.8 ± 2.9c	340.2 ± 7.9c	83.8 ± 4.3c	534.7 ± 8.3c	97.8 ± 7.7c	575.7 ± 19.8d	214.1 ± 18.1c
	Medium strips	11.8 ± 1.3	106.8 ± 5.3	45.3 ± 2.4bc	411.1 ± 12.7b	90.6 ± 5.4c	580.5 ± 9.4b	115.5 ± 8.7bc	641.4 ± 35.3c	245.9 ± 20.2bc
	Wide strips	14.1 ± 1.4	114.7 ± 5.0	52.0 ± 2.9ab	438.6 ± 13.8ab	108.5 ± 2.3b	605.5 ± 13.0b	133.7 ± 12.1b	801.1 ± 46.0b	286.7 ± 19.5b
	Sole wheat	15.0 ± 1.3	120.6 ± 2.8	56.7 ± 2.1a	480.9 ± 21.8a	128.1 ± 6.0a	724.8 ± 3.6a	192.9 ± 23.8a	959.1 ± 42.9a	415.3 ± 17.3a
	LSD	5.7	21.2	8.8	52.7	14.6	28.5	29.1	48.9	50.9
2019–20	Narrow strips	10.1 ± 1.3^NS^	102.5 ± 8.3^NS^	41.2 ± 2.4c	346.1 ± 8.5c	89.1 ± 2.6b	550.3 ± 9.2c	102.2 ± 7.9b	596.9 ± 20.7d	227.6 ± 18.6b
	Medium strips	10.6 ± 1.4	108.7 ± 5.5	47.3 ± 1.2bc	419.4 ± 13.2b	91.4 ± 3.2b	598.2 ± 9.7b	118.2 ± 9.1b	665.3 ± 38.2c	248.1 ± 20.4b
	Wide strips	12.8 ± 1.5	116.2 ± 4.7	52.1 ± 3.0ab	446.3 ± 15.1ab	101.8 ± 4.0b	624.1 ± 13.5b	126.8 ± 12.6b	831.4 ± 47.9b	275.7 ± 15.3b
	Sole wheat	13.8 ± 1.4	123.0 ± 2.9	56.9 ± 2.2a	460.8 ± 21.4a	124.6 ± 13.7a	746.7 ± 24.5a	198.8 ± 19.2a	995.3 ± 32.4a	424.4 ± 17.1a
	LSD	5.4	21.5	8.5	53.7	26.1	42.0	25.7	47.5	51.3
2020–21	Narrow strips	8.2 ± 1.2^NS^	94.9 ± 8.0^NS^	37.0 ± 2.3c	326.5 ± 8.3c	78.4 ± 2.5b	511.1 ± 37.6c	89.1 ± 9.9c	551.9 ± 19.8d	200.1 ± 21.9c
	Medium strips	8.4 ± 0.9	100.8 ± 5.2	42.9 ± 4.1bc	397.1 ± 12.6b	80.7 ± 3.0b	557.1 ± 25.4c	104.4 ± 8.7c	617.5 ± 35.2c	220.5 ± 25.4bc
	Wide strips	10.8 ± 1.4	108.0 ± 4.5	47.5 ± 2.8ab	422.9 ± 14.5ab	101.2 ± 5.4a	608.6 ± 16.4b	136.0 ± 15.2b	776.9 ± 45.9b	269.7 ± 13.2b
	Sole wheat	11.7 ± 1.3	114.5 ± 2.7	52.1 ± 2.1a	436.8 ± 21.2a	112.6 ± 7.7a	699.7 ± 31.9a	181.5 ± 23.7a	934.2 ± 43.1a	389.2 ± 18.5a
	LSD	4.6	20.7	11.3	52.4	16.7	96.4	29.7	49.0	50.8

Means are averages over three replicates ± standard error of the mean. NS: Non-significant differences were detected between means using the LSD test.

**Table 2:** Dry matter distribution in soybean at 45, 75, and 105 days after seed emergence (DAS) and maturity under different planting systems.

Years	Planting systems	Soybean dry matter distribution (g m^-2^)								
		45 DAS		75 DAS		105 DAS		Maturity		
		Root	Straw	Root	Straw	Root	Straw	Root	Straw	Grains
2018–19	Narrow strips	6.5 ± 1.9b	76.9 ± 8.8b	20.1 ± 2.5c	120.5 ± 14.9c	24.1 ± 2.9c	170.2 ± 15.1c	40.2 ± 8.3c	281.3 ± 34.5c	50.1 ± 5.0c
	Medium strips	7.8 ± 1.8b	87.3 ± 7.4b	26.7 ± 3.8b	160.2 ± 22.8b	32.4 ± 4.7b	232.6 ± 27.4bc	53.4 ± 9.4b	373.9 ± 53.1b	61.3 ± 6.7bc
	Wide strips	8.2 ± 1.6b	104.4 ± 9.1ab	29.7 ± 3.5ab	178.4 ± 20.9ab	35.7 ± 4.2ab	273.5 ± 30.2b	59.5 ± 13.0b	416.2 ± 48.7ab	74.5 ± 10.1b
	Sole soybean	10.1 ± 1.1a	136.1 ± 10.7a	34.3 ± 2.0a	212.6 ± 18.4a	41.2 ± 2.4a	377.1 ± 22.6a	68.6 ± 3.6a	480.5 ± 27.5a	92.1 ± 16.0a
	LSD	1.9	33.1	5.9	35.6	7.4	90.3	11.7	82.1	15.9
2019–20	Narrow strips	7.2 ± 1.9b	77.4 ± 10.4b	22.5 ± 1.5d	101.3 ± 8.6c	35.2 ± 3.1c	184.1 ± 31.3b	54.3 ± 9.7c	218.6 ± 20.3c	53.8 ± 7.9c
	Medium strips	8.9 ± 1.8ab	92.1 ± 10.3b	26.7 ± 3.1c	153.6 ± 19.5bc	39.6 ± 3.0c	256.8 ± 37.2b	69.2 ± 5.0bc	321.7 ± 29.1bc	64.7 ± 12.5bc
	Wide strips	9.2 ± 1.6ab	114.5 ± 14.8ab	31.2 ± 3.6b	203.4 ± 13.6b	49.8 ± 4.7b	317.3 ± 35.9b	87.3 ± 10.6b	385.2 ± 64.4ab	78.1 ± 9.9b
	Sole soybean	11.6 ± 2.7a	145.8 ± 15.6a	38.3 ± 3.2a	321.2 ± 35.8a	57.7 ± 4.6a	486.5 ± 40.6a	121.4 ± 11.3a	452.8 ± 34.4a	100.3 ± 17.0a
	LSD	2.8	39.6	4.1	85.3	7.3	140.8	22.9	126.8	18.9
2020–21	Narrow strips	8.4 ± 2.1b	86.1 ± 9.3b	24.1 ± 2.2c	118.6 ± 6.5c	34.5 ± 3.0c	208.8 ± 30.1b	55.2 ± 7.2c	260.7 ± 6.8c	57.9 ± 9.3c
	Medium strips	9.7 ± 1.9b	97.6 ± 9.8b	29.8 ± 3.6b	166.5 ± 18.4bc	40.2 ± 2.6c	276.4 ± 34.4b	70.5 ± 4.6bc	365.9 ± 39.1bc	68.6 ± 12.4bc
	Wide strips	10.2 ± 1.9ab	118.7 ± 12.7ab	34.2 ± 4.1b	203.6 ± 14.1b	49.6 ± 4.8b	333.6 ± 38.1b	85.9 ± 9.5b	425.4 ± 62.5ab	84.1 ± 11.1b
	Sole Soybean	12.6 ± 2.7a	153.6 ± 17.6a	40.3 ± 2.9a	310.3 ± 26.9a	57.9 ± 5.2a	504.3 ± 40.4a	114.3 ± 9.1a	497.3 ± 34.5a	105.2 ± 17.8a
	LSD	2.7	38.3	4.8	70.6	6.2	137.8	19.9	109.5	18.5

Means are averages over three replicates ± standard error of the mean.

### Nitrogen uptake of wheat and soybean

**Figure 3:**
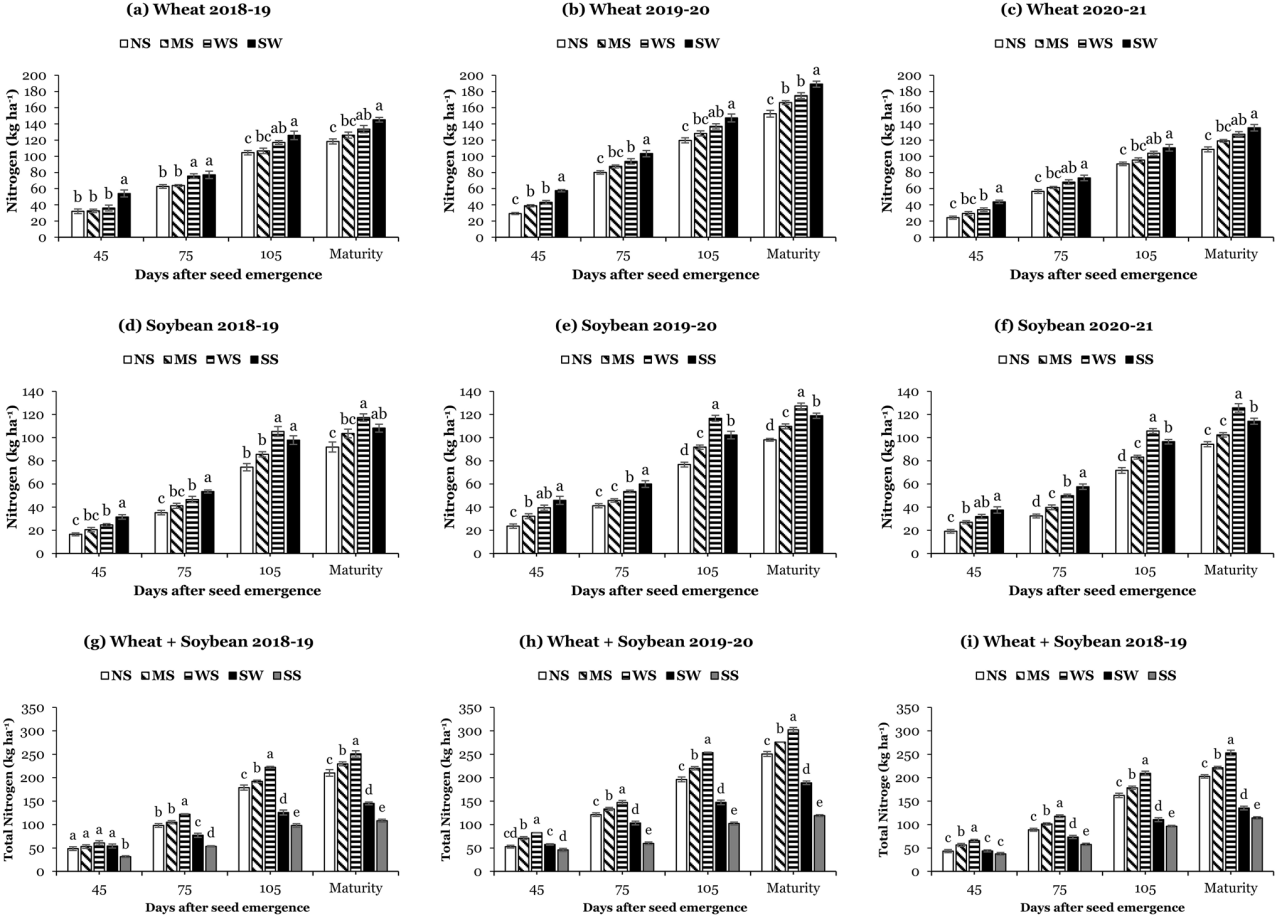
Nitrogen uptake of wheat (**a**–**c**) and soybean (**d**–**f**), and total nitrogen uptake (**g**–**i**) at 45, 75, and 105 days after seed emergence and maturity under different planting systems: SW (sole wheat), SS (sole soybean), NS (narrow strips; 0.9 m), MS (medium strips; 1.8 m), and WS (wide strips; 2.7 m). Means are averages over three replicates ± standard error of the mean.

Nitrogen uptake in wheat and soybean was significantly affected by the different planting systems at all sampling times, and data are given in Fig. 3. Average, over the years, at all sampling times, wheat accumulated significantly higher nitrogen in sole wheat than in intercropping systems (Fig. 3a–c). In contrast, in WSI, the mean maximum nitrogen uptake was noticed in wide strips, while the mean minimum nitrogen uptake was observed in narrow strips, indicating that the higher nitrogen uptake is directly associated with improved wheat growth in intercropping systems. Whereas at 45 and 75 DAS, the highest nitrogen uptake was obtained in sole soybean than in intercropping systems, while at 105 DAS and maturity, the pattern of nitrogen uptake was changed, and the highest nitrogen uptake was measured in wide strips than sole soybean (Fig. 3d–f). On average, at maturity, the highest nitrogen uptake in wheat (156.4 kg ha^−1^) and soybean (123.4 kg ha^−1^) was measured in sole wheat and wide strips, respectively, and the lowest nitrogen uptake of wheat (126.4 kg ha^−1^) and soybean (94.8 kg ha^−1^) was obtained in sole wheat and sole soybean, respectively. Moreover, at all sampling times, intercropping systems accumulated significantly higher total nitrogen (wheat nitrogen uptake + soybean nitrogen uptake) than the corresponding values in sole systems (Fig. 3g–i). For instance, at maturity, the total nitrogen uptake of narrow strips (221.2 kg ha^−1^), medium strips (242.3 kg ha^−1^), and wide strips (268.6 kg ha^−1^) was 72%, 55%, and 41% higher than sole wheat and 136%, 113%, and 94% higher than sole soybean, suggesting that the due to spatial and temporal complementarity intercropping systems accumulated more nitrogen than sole systems.

### Yield and yield components of wheat and soybean

**Figure 4:**
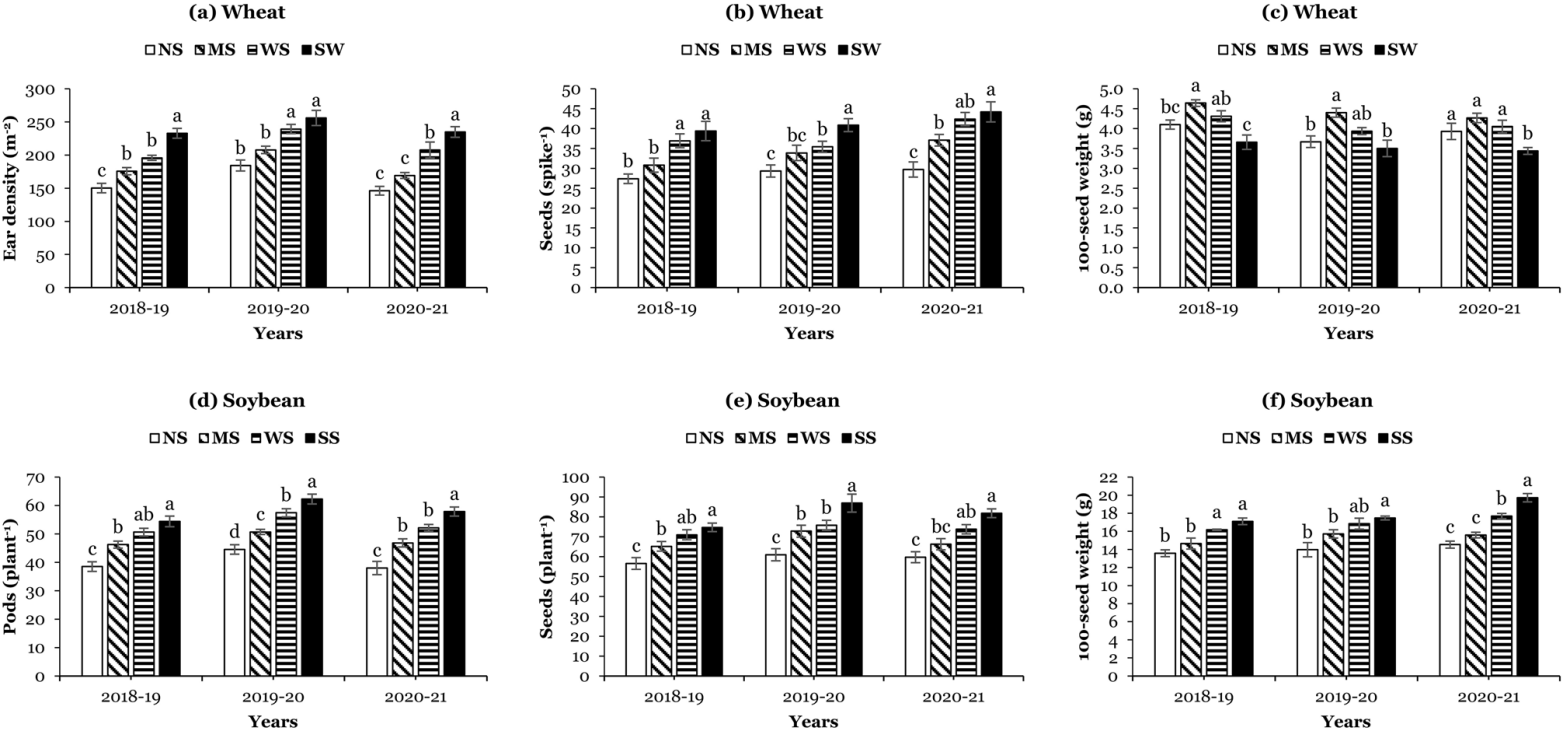
Ear density per meter square (**a**), seeds per spike (**b**), and 100-seed weight of wheat (**c**); pods per plant (**d**), seeds per plant (**e**), 100-seed weight (**f**) of soybean in 2018–19, 2019–20, and 2020–21 under different planting systems: SW (sole wheat), SS (sole soybean), NS (narrow strips; 0.9 m), MS (medium strips; 1.8 m), and WS (wide strips; 2.7 m). Means are averages over three replicates ± standard error of the mean.

Yield and yield components of wheat differed significantly in all planting systems, as presented in (Table 3; Fig. 4a–c). Sole wheat always had a significantly higher grain yield than in intercropping systems. However, in WSI, the wide strips had a higher grain yield than the narrow and medium strips. For instance, the wide strips increased the wheat grain yield by 14% and 9% compared to narrow strips and medium strips, respectively. Moreover, in yield components, the ear density m^2^ and seeds spike^-1^ of sole wheat were significantly higher than those in intercropping systems. In contrast, the hundred-seed weight of intercropped wheat was significantly higher in intercropping than sole wheat. On average, in WSI, the wide strips had higher ear density (214.2 m^2^) and seeds (38.2 spike^−1^) of wheat than narrow and medium strips, and the maximum hundred seed weight (4.4 g) of wheat was observed in medium strips than wide and narrow strips, suggesting that wheat in wide and medium strips invested their photosynthates more efficiently for yield and yield components than in narrow strips, especially at the time of the formation of yield components.

**Table 3:** Grain yield, land equivalent ratio, and the competitive ratio of wheat and soybean under different planting systems.

Years	Planting systems	Grain yield (kg ha^-1^)			Land equivalent ratio (LER)			Competitive ratio	
		Wheat	Soybean	Total grain yield	Wheat	Soybean	Total LER	Wheat	Soybean
2018–19	Sole wheat	4518.8 ± 171.8a	–	–	–	–	–	–	–
	Sole soybean	–	1689.1 ± 45.7a	–	–	–	–	–	–
	Narrow strips	2533.3 ± 206.5b	914.4 ± 54.9c	3447.7 ± 260.1b	0.56 ± 0.05b	0.54 ± 0.05c	1.11 ± 0.09b	1.04 ± 0.04a	0.97 ± 0.04b
	Medium strips	2622.2 ± 190.7b	993.6 ± 74.6c	3615.8 ± 264.7b	0.58 ± 0.05b	0.59 ± 0.05b	1.17 ± 0.10b	0.99 ± 0.04a	1.01 ± 0.04b
	Wide strips	2857.1 ± 173.4b	1206.6 ± 40.2b	4063.0 ± 212.8a	0.64 ± 0.05a	0.72 ± 0.04a	1.35 ± 0.09a	0.88 ± 0.04b	1.13 ± 0.05a
	LSD	485.7	174.1	183.2	0.04	0.04	0.07	0.06	0.06
2019–20	Sole wheat	4301.1 ± 115.7a	–	–	–	–	–	–	–
	Sole soybean	–	1596.3 ± 38.8a	–	–	–	–	–	–
	Narrow strips	2307.7 ± 105.1b	836.1 ± 68.9c	3143.7 ± 173.3b	0.54 ± 0.03b	0.52 ± 0.05c	1.06 ± 0.07b	1.03 ± 0.04a	0.97 ± 0.04b
	Medium strips	2441.4 ± 147.6b	915.8 ± 64.5bc	3357.2 ± 211.2b	0.57 ± 0.03ab	0.57 ± 0.05b	1.14 ± 0.08b	0.99 ± 0.02a	1.01 ± 0.02b
	Wide strips	2646.6 ± 228.7b	1125.3 ± 84.6b	3771.9 ± 312.5a	0.62 ± 0.06a	0.71 ± 0.06a	1.32 ± 0.11a	0.87 ± 0.01b	1.15 ± 0.01a
	LSD	375.3	159.9	300.8	0.06	0.03	0.09	0.10	0.11
2020–21	Sole wheat	4231.6 ± 207.9a	–	–	–	–	–	–	–
	Sole soybean	–	1641.7 ± 88.3a	–	–	–	–	–	–
	Narrow strips	2197.5 ± 117.4c	858.5 ± 30.6c	3055.0 ± 146.4c	0.52 ± 0.01c	0.53 ± 0.03c	1.04 ± 0.03c	0.99 ± 0.04a	1.01 ± 0.04c
	Medium strips	2305.3 ± 123.7c	944.3 ± 44.5c	3249.6 ± 166.1b	0.54 ± 0.01b	0.58 ± 0.03b	1.12 ± 0.04b	0.95 ± 0.04a	1.06 ± 0.05b
	Wide strips	2523.4 ± 133.7b	1146.5 ± 39.6b	3669.9 ± 173.0a	0.60 ± 0.01a	0.70 ± 0.03a	1.30 ± 0.04a	0.85 ± 0.02b	1.17 ± 0.03a
	LSD	168.9	139.2	57.6	0.01	0.02	0.02	0.05	0.05

Means are averages over three replicates ± standard error of the mean. NS: Non-significant differences were detected between means using the LSD test.

An increase in strip width significantly increased the soybean yield in all intercropping systems, but the sole soybean always had a significantly higher yield than intercropped soybean yield in narrow, medium, and wide strips (Table 3; Fig. 4d–f). Over the years, the intercropped soybean had 53%, 58%, and 71% of sole soybean yield in narrow strips, medium strips, and wide strips, respectively. Furthermore, in all years, the pods plant^−1^, seeds plant^−1^, and hundred seed weight of soybean were significantly lower under intercropping systems than the corresponding values in sole soybean. Compared to narrow strips, the wide strips increased the pods plant^−1^ (by 32%), seeds plant^−1^ (by 24%), and hundred seed weight (by 20%), indicating that the improvement in yield and yield components of intercropped soybean in wide strips were due to the relaxed competitive interactions between wheat and soybean. Additionally, in WSI, the total grain yield of wide strips (3835.2 kg ha^−1^) was significantly higher than medium strips (by 13%; 3407.5 kg ha^−1^) and narrow strips (by 19%; 3215.8 kg ha^−1^), demonstrating that the use of wide strips was more effective and efficient in utilizing the available resources (land and nitrogen) than medium and narrow strips in WSI (Table 3; Fig. 4).

### Land equivalent ratio and competition ratio

Different strip widths in WSI significantly affected the pLERw, pLERs, and total LER values (Table 3). On average, among WSI, wheat and soybean had the highest and lowest pLERw and pLERs values in wide strips and narrow strips, respectively. The values of total LER in narrow strips, medium strips, and wide strips ranged from 1.04 to 1.35, exhibiting a land use advantage over sole systems. Generally, across the years and WSI, the mean values of total LER were higher in wide strips than in narrow and medium strips. Wide strips increased the total LER by 24% and 16% compared to narrow and medium strips, respectively, demonstrating that wide strips were more advantageous in achieving the intercropping benefits. Moreover, the changes in strip width significantly impacted the competitive abilities of wheat and soybean in WSI (Table 3). The mean maximum (1.02) and minimum (0.87) values of CRw were noted in narrow strips and wide strips, while the opposite trends were noticed for the values of CRs, and the average highest (1.15) and lowest (0.98) values of CRs were noticed in wide strips and narrow strips, respectively. Overall, the wide strips increased the CRs by 17% and reduced the CRw by 12% compared to narrow strips, exhibiting that the competitive ability of intercrops in the WSI was closely associated with changes in strip width.

### Economic viability

Grain yields of both crops impacted the gross income, benefit-to-cost ratio, and net profit of all systems (Table 4). The mean maximum gross income (1813 US$ ha^−1^) and net profit (1081 US$ ha^−1^) were recorded in wide strips, while the mean minimum gross income (1075 US$ ha^−1^) and net profit (320 US$ ha^−1^) were observed in sole soybean. Average across the years and planting systems, the wide strips increased the net profit by 34% and 42% compared to narrow strips and sole wheat, respectively, suggesting that the strip intercropping of wheat and soybean was economically advantageous over narrow strips in WSI and sole wheat. Moreover, the benefit-to-cost ratio differed considerably among various planting systems; the average highest (2.6) benefit-to-cost ratio was noticed in wide strips, whereas the average lowest (1.5) benefit-to-cost ratio was noted in sole soybean.

**Table 4:** Total expense, gross income, net profit, and the benefit-to-cost ratio of wheat and soybean under strip intercropping and sole cropping systems.

Planting systems	Total expense (US$ ha^-1^)			Gross income (US$ ha^-1^)			Net profit (US$ ha^-1^)			Benefit-to-cost ratio		
	2018–19	2019–20	2020–21	2018–19	2019–20	2020–21	2018–19	2019–20	2020–21	2018–19	2019–20	2020–21
Sole wheat	613	789	1026	1307	1418	1839	694	628	813	2.1	1.8	1.8
Sole soybean	555	712	927	1299	1038	991	744	325	64	2.3	1.5	1.1
Narrow strips	579	743	965	1548	1447	1655	969	704	690	2.7	1.9	1.7
Medium strips	588	743	935	1630	1533	1744	1042	790	809	2.8	2.1	1.9
Wide strips	613	789	1026	1849	1723	1942	1235	933	916	3.0	2.2	1.9

The local market price for wheat was US$ 233 t^-1^ in 2019, 258 t^-1^ in 2020, and 345 t^-1^ in 2021, and for soybean was US$ 697 t^-1^ in 2019, 650 t^-1^ in 2020, and 615 t^-1^ in 2021.

## Discussion

Intercropping contains multifaceted research interests from a broad range of researchers, including agronomists, agroecologists, and environmentalists. Despite the wide range of agronomic advantages and ecological services offered by intercropping, large-scale farmers still prefer mechanized monocultures, where crops mature evenly with improved methods of plant protection and crop varieties over un-mechanized intercropping systems, where intercrops mature unevenly and do not have proper plant protection measures and intercropping-specific crop varieties^45–47^. Thus, resource-exhaustive monocultures requiring higher anthropogenic inputs exert extra pressure on the ecosystem compared to intercropping systems that have a lower environmental impact. This situation demands us to investigate such spatio-temporal attributes of intercropping systems that could compete with monocropping systems in terms of mechanization and food security to extend their ecological advantages. Subsequently, with an objective to explore the potential spatio-temporal arrangements for possible mechanization of intercropping systems with higher agro-economic and ecological returns, we conducted this study to verify our three hypotheses. The first hypothesis was confirmed by our data; compared to narrow strips, both intercrops had a higher dry matter and grain yield with wide strips. The data strongly confirmed the second hypothesis: intercropping systems accumulated higher total nitrogen than sole systems. The third hypothesis was also strongly confirmed; both intercrops were planted, harvested, and threshed easily with the existing farm machinery in wide strips. This ultimately increased the net profit by saving considerable labour expenses on sowing, harvesting, and threshing, reducing the yield losses with manual management of crops. Eventually, our findings revealed that wide strips for intercrops are (a) more appropriate and favourable for obtaining the maximum benefits of WSI, (b) critical for land productivity and large-scale adoption of intercropping systems, and (c) intercropping systems could play an important role in decreasing the environmental impact of agriculture as legume-based intercropping systems require fewer anthropogenic inputs (nitrogen and phosphorus) than sole cereal systems.

Our results demonstrated that changing the strip widths in WSI affected the growth of intercropped species, which might be linked with competition between intercrops for available resources^12,48^; as the competitive ability of intercrops positively or negatively influenced by the changes in the spatial arrangement of crops in intercropping^10,20^. Similar to our results, a recent review reported a close association between strip widths and interspecific interactions of intercrops in intercropping^40^. The increase in width in the present study shifted the impact of spatial arrangement in favour of soybean and increased the overall yield and mechanization potential of the WSI. Besides mechanization, competition for sunlight is another important constraint, especially in narrow strips, that limits the production of cereal/legume intercropping systems because cereals often provide shade to legumes^41,42^, as observed in this study that early-planted wheat (47 ± 06 Days) intercepted more sunlight due to its greater leaf area and plant height, which affected the initial growth and competitive ability of soybean for water and nutrient uptake, particularly in narrow strips. However, widening the strip width from 0.9 to 2.7 m increased the leaf area and dry matter of soybean, indicating that the wider strips helped to establish an ecological niche that relaxed the competition for sunlight, water, and nutrients between intercrops. These results are consistent with past research in which scientists had confirmed that the intercrops attained the highest values of leaf area index, dry matter, and grain yield in wide strips than in narrow strips^10,31,35,38,49^.

Data from this study further revealed that intercropping with wide strips was more advantageous than with narrow strips. The higher dry matter of intercrops in wide strips demonstrates the efficient utilization of available resources^5^. This could be associated with a high light interception by intercrops because it is directly proportional to the leaf area^39,41^. Although the values of the partial leaf area of intercrops in wide strips were lower than the corresponding values in pure stands, however, the total LAI in wide strips was consistently higher than the LAI of sole wheat or soybean, which could enhance the radiation use efficiency of WSI. This is consistent with past reports ^50,51^, in which they obtained higher grain yield with wide strips than narrow strips in cereal/legume strip intercropping systems and linked it with an increased radiation use efficiency of intercrops^20,52^. The diversity in intercropping leads to better ecological complementarity due to less niche overlap and variable competitive ability^53^. The cereals usually have a competitive advantage over legumes, and incompatible strip width may intensify this competition. On the other hand, provided the suitable spatio-temporal arrangements, the cereal-legume intercropping may also complement each other. For instance, in the present study, the reduced competition in wider strips helped to complement the temporal difference and nitrogen uptake and utilization in wheat and soybeans. Firstly, without interspecific competition, the early-sown wheat showed vigorous initial growth and nitrogen uptake to invest in its reproductive parts at later growth stages. Secondly, the wide strips reduced the competitive pressure of wheat over soybean and facilitated interspecific complementarity during the co-growth period. For example, the anthesis of wheat synchronized with the flowering stage of soybean and higher competitive advantage of border-row wheat plants induced higher nitrogen fixation in soybean due to exhaustive uptake of nitrogen from border rows (supported by better light availability). Previous literature has verified the strong recovery growth of soybeans after the harvesting of cereal crops^17^. Similarly, our results also showed the growth improvement in soybeans after the harvesting of wheat; in addition, the extra growing space for soybeans in wide strips than narrow strips led to better resource use and the formation of yield components and yield. Altogether, the higher total nitrogen uptake in intercropping systems with the same nitrogen inputs verified the better niche complementarity and less environmental impact of the wheat/soybean relay intercropping systems over their sole systems^54,55^. Furthermore, the highest total nitrogen uptake in wide strips of wheat/soybean relay intercropping systems verified that wider strips complement the temporal differences between wheat and soybean in obtaining yield and ecological advantages.

The higher yield advantage in wide strips verified the ecological benefits of increased strip width and confirmed that the changing planting configuration, manipulated by strip widths, had a direct impact on the yield and yield components of intercrops in WSI. For instance, the grain yield of intercropped wheat and soybean considerably increased from 54 and 53% (planted in narrow strips, where both intercrops were sown in narrow strips of 0.9 m) to 62% and 71% (grown in wide strips, where both intercrops were planted in wide strips of 2.7 m), respectively. The higher grain yield of intercrops in wide strips was mainly gained from increased dry matter accumulation and its investment in yield formation components of wheat (spike m^−2^, by 34%; seeds spike^−1^, by 33%; and hundred seed weight, by 5%) and soybean (pod plant^−1^, by 32%; seed plant^−1^, by 24%; and hundred seed weight, by 20%) than narrow strips, whereas a decrease in spike m^−2^, seeds spike^−1^, and hundred seed weight of wheat, and pod plant^−1^, seed plant^−1^, and hundred seed weight of soybean largely caused the yield loss of intercrops narrow strips compared to wide strips. This was the implication of the functional complementary and facilitative effects between intercrops^8^, as the negative effects of wheat shade on soybean were reduced because the soybean border rows were farthest from wheat border rows in wide strips than narrow strips^39^, which substantially increased the soybean yield while maintaining wheat yield. Additionally, in wide strips, the intra-specific competition for growing space and resources was also lessened due to a temporal niche differentiation; for instance, the wheat attained the anthesis and grain filling stages earlier than the flowering and pod formation stages of soybean, this temporal difference allowed both crops to use land and other resources more efficiently than sole systems, resulting in a relative yield advantage for soybean and wheat. All in all, for land use in WSI, we can conclude that intercrops in wide strips produced more grain per unit area of land than in narrow strips, which confirmed the benefits of intercropping over sole systems.

Overall, the positive impacts of wide strips on LER were significant in all years of this study. Compared to narrow strips, the increased LER with wide strips was mainly attributed to ecological niche optimization provided by the edge row and spatial light distribution advantage. Our findings and previous studies on cereal/legume intercropping^31,36^ indicate the positive impact of wide strips on LER^56,57^. Importantly, wide strips in intercropping can be operated and managed using the existing small farm equipment, especially in developing countries (Pakistan and India), where farmers do not have large sowing and harvesting machines as farmers have in Europe or the USA. Consequently, with small farm machinery and wide strips in WSI, it is easy for farmers to achieve the economic, yield and environmental benefits of intercropping. These results highlight the previously mentioned concerns that if researchers do not design new wide-strip intercropping systems or develop small farm machinery, traditional intercropping will become less profitable for farmers. In conclusion, the wide strips are easier to manage, require less labor work, and produce higher grain yields than narrow strip designs in intercropping, as we observed in this study; therefore, we have to replace the narrow strip intercropping designs with wide strip intercropping systems.

Previously, many researchers have confirmed that intercropping with 2 m strips produced higher net profit than 3 or 4 m^1,12,20,40^. However, in this study, the wide strips of 2.7 m gave higher net profit than the narrow strips of 0.9 m; results are similar to those of previous studies^4,31,35–38^. A higher net profit of wide strips indicated that wheat and soybean could be planted and harvested using the existing small farm machinery. The significant improvement in the net profit of wide strips was largely attributed to a greater relative grain yield of soybean with a maintained wheat relative grain yield, which greatly contributed to increasing the net profit of wide strips over narrow strips because soybean was valued at four times more expensive than the value of wheat. Importantly, in all years of this study, compared to narrow strips, farm machinery saved 212 US$ per season in 2019, 240 US$ per season in 2020, and 300 US$ per season in 2021 in wide strips, which is a huge net profit for the farmers of developing countries, e.g., Pakistan, where the average monthly income is just around 100 ± 10 US$ per season^58^. The practical implications of our study are clear; intercropping with wide strips is the better planting strategy for producing legumes and cereals in a sustainable and environmental-friendly way with limited land and fewer anthropogenic inputs. However, future research is needed to fully understand the water, light, and nutrient utilization mechanism of intercrops in wide strips under intercropping systems.

## Conclusion

The study data confirmed that the wide strips produced higher relative grain yields and saved 20%-30% of the land than narrow strips in WSI or sole systems. Notably, the net profit of wide strips was greatly higher than the net profits of medium strips and narrow strips in WSI; it was also higher than the net profits of sole wheat and sole soybean. Moreover, the intercropping systems accumulated more nitrogen from the soil profile than sole systems, which showed the advantage of intercropping systems over sole cropping systems for saving fertilizers (nitrogen) and the environment. Besides, narrow strips in intercropping systems are difficult to manage because most of the farm machinery has been developed for homogeneous and large cultivated areas. Therefore, the narrow strips need to be transformed into wide strips that could be mechanized and tailored using the existing farm machinery without losing the crop diversification advantage of intercropping. All in all, these results support the great potential of wide strip intercropping systems for diversifying and restoring the exhaustive sole systems, which could contribute to the sustainable intensification of agriculture. However, without addressing the labor challenge, i.e., through mechanization or spatial management of intercrops, it is difficult for researchers and policymakers to promote the adoption of cereal legume intercropping systems.

## Materials and methods

### Ethics statement

No specific permissions were required to conduct these field experiments. All experiments were performed according to institutional guidelines of the Islamia University of Bahawalpur, Pakistan. Besides, it is confirmed that all methods were performed following the relevant guidelines/regulations/legislation.

### Research site description

**Figure 5:**
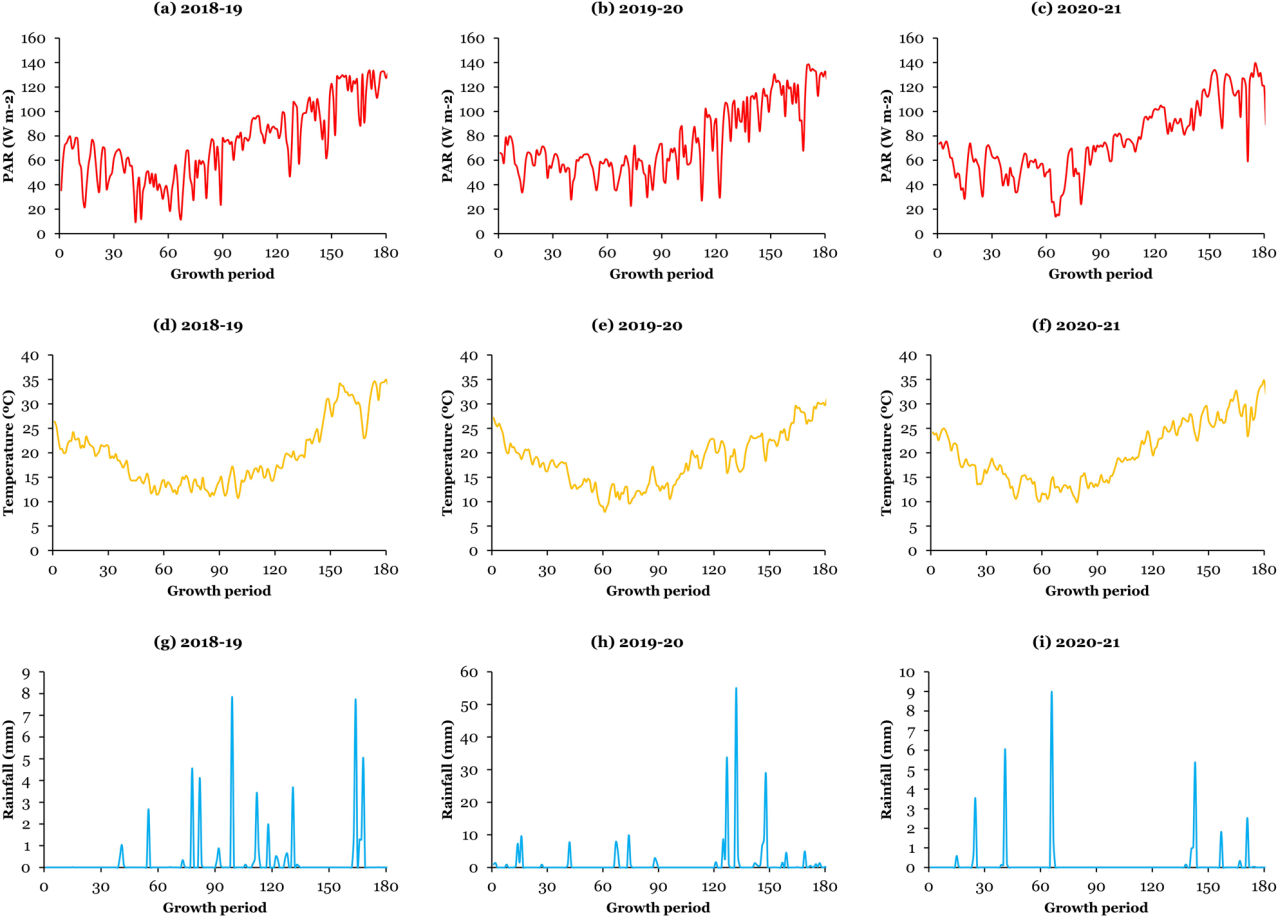
Daily photosynthetically active solar radiation (PAR; (**a**) for 2018–19, (**b**) for 2019–20, (**c**) for 2020–21), temperature ((**d**) for 2018–19, (**e**) for 2019–20, (**f**) for 2020–21), and rainfall ((**g**) for 2018–19, (**h**) for 2019–20, (**i**) for 2020–21) during the growing season of wheat.

These experiments were carried out during the winter and spring seasons of 2018–19, 2019–20, and 2020–21 at Khairpur Tamewali under arid irrigated conditions, the experimental field of the National Research Center of Intercropping (29.57°N, 72.25°E; altitude of 130 m), the Islamia University of Bahawalpur, Pakistan. The study area is located in the south of Pakistan, 60 km southeast of Bahawalpur Division, South Punjab. The research region has an annual rainfall of 145 mm (typically, most rain occurs during the monsoon season from end-June to end-August) with a mean temperature of 25.7 °C. The soil type is sandy loam with organic matter of 5.3 g kg^−1^, pH of 7.9, total nitrogen of 0.4 g kg^−1^, total available phosphorus of 5.3 mg kg^−1^, total available potassium of 78.9 mg kg^−1^, and bulk density of 1.47 Mg m^−3^. The daily PAR, average temperature, and total precipitation during the three growing seasons are presented in Fig. 5. In addition, the total rainfall from sowing to harvesting (November to April) of crops was 53 mm, 232 mm, and 34 mm in 2018–19, 2019–20, and 2020–21, respectively.

### Experimental design and crop management

These experiments were carried out using a randomized complete block design with three replications. In total, there were five systems: three strip intercropping systems differing with strip widths (narrow strips, 0.9 m strip for each intercrop; medium strips, 1.8 m strip for each intercrop; and wide strips, 2.7 m strip for each intercrop) and two sole crop systems (sole wheat and sole soybean). The complete planting configurations, e.g., row-to-row distance, strip distance between wheat and soybean strips, plant-to-plant distance, number of rows of wheat and soybean in each strip, and the overall planting density of each treatment, are presented in Table 5. For the validity of results, we conducted this field study on an area of one hectare (100 m × 100 m); the size of each experimental plot in narrow strips, medium strips, wide strips, sole wheat, and sole soybean was 388.8 m^2^ (21.6 m × 18 m), 777.6 m^2^ (43.2 m × 18 m), 1166.4 m^2^ (64.8 m × 18 m), 432 m^2^ (24 m × 18 m), and 432 m^2^ (24 m × 18 m), respectively. Planting, harvesting, and threshing were done manually in narrow and medium strips. While in wide strips, sole wheat, and sole soybean, planting, harvesting and threshing were done using the Rabi-Drill and Combine-Harvester, respectively. All other operations were kept uniform in all systems.

**Table 5:** The planting configuration of wheat and soybean under different planting systems.

Planting systems	Row distance			Strip distance*	Plant distance		Number of rows		Overall plant density **		Total ***
	(cm)			(cm)	(cm)		(m^-1^)		(plants m^-2^)		
	Wheat	Soybean		Wheat/Soybean	Wheat	Soybean	Wheat	Soybean	Wheat	Soybean	
Sole wheat	15	30		–	–	–	6.67	–	220	–	220
Sole soybean	15	30		–	–	20	–	3.33	–	16.7	16.7
Narrow strips	15	30		22.5	–	10	3.33	1.67	110	16.7	127.8
Medium strips	15	–		22.5	–	10	3.33	1.67	110	16.7	128.8
Wide strips	–	30		22.5	–	10	3.33	1.67	110	16.7	128.8

*Distance between the strips of wheat and soybean in intercropping systems. **Sole wheat and soybean were sown according to the local planting densities: 2,200,000 plants ha^-1^ for wheat and 167,000 plants ha^−1^ for soybean. However, in strip intercropping systems, we used 50% of sole wheat density for intercropped wheat and 100% of sole soybean density for intercropped soybean, rendering the relative density of intercropped wheat and soybean equal to 0.5 and 1, respectively. *** The total planting density in strip intercropping systems was equal to 1.5; therefore, the design of strip intercropping systems was additive.

**Figure 6:**
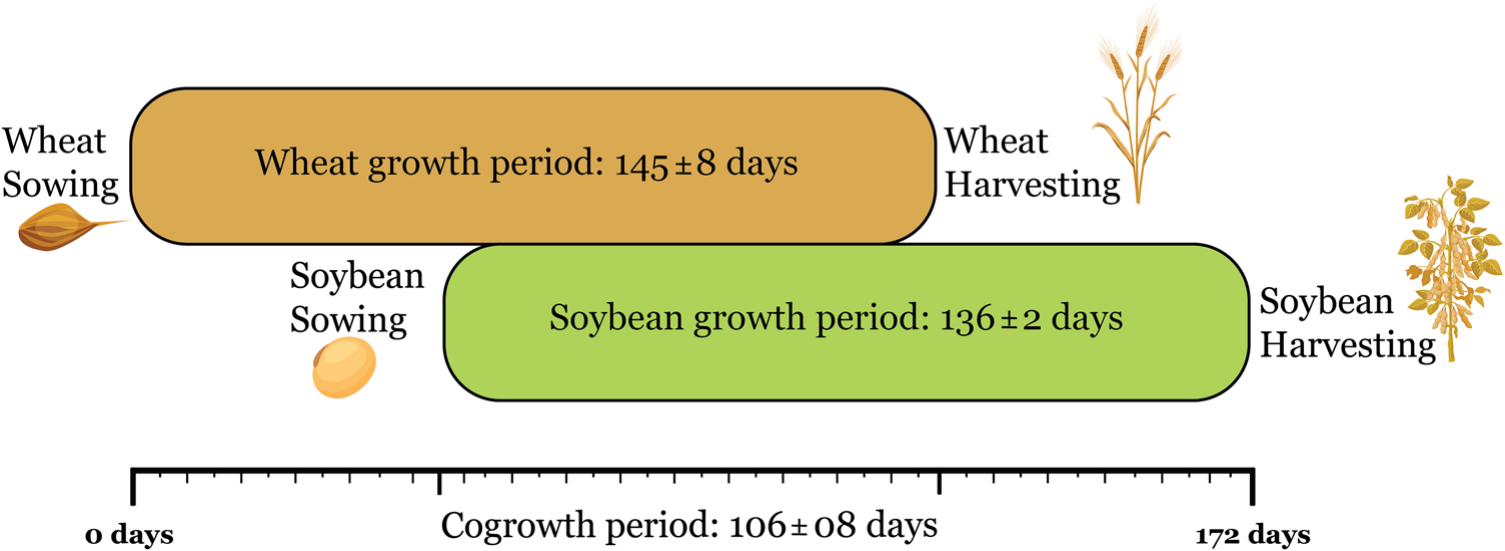
The growth period of wheat and soybean under strip intercropping systems. The upper bar represents the wheat growing period (first sown intercrop species), and the lower bar represents the soybean growing period (second planted intercrop species). The co-growth period is the number of days when both crops grow together.

Wheat variety ‘Faisalabad-2008’ was sown on November 10, 15, and 17 in 2018, 2019, and 2020, respectively, while the determinate soybean variety ‘NARC-2’ was sown on December 21, 23, and 25 in 2018, 2019, and 2020, respectively. Wheat was harvested on April 12, 09, and 02 in 2019, 2020, and 2021, respectively, while soybean was harvested on May 07, 05, and 02 in 2019, 2020, and 2021, respectively. Overall, on average, across the years, the total crop growth period of the WSI was 172 ± 06 days, the total days of wheat and soybean growing period were 145 ± 08 days and 136 ± 02 days, respectively, and the total co-growth period was 106 ± 08 days (Fig. 6). At the time of wheat and soybean sowing, phosphorus was applied @ 60 kg ha^−1^. The first dose of nitrogen was applied @ 60 kg ha^−1^ to wheat strips, and 30 kg ha^−1^ to soybean strips when wheat was at the tillering stage^59^ and soybean was at the fifth trifoliate stage^60^. The second nitrogen dose was applied @ 60 kg ha^−1^ to wheat strips, and 30 kg ha^−1^ to soybean strips when wheat was at the booting stage^59^ and soybean was at the R_2_ stage^60^. Urea and diammonium phosphate were used as a source of nitrogen and phosphorus, respectively.

### Measurements

In all years of this study, the leaf area index (LAI) of both crops was determined three times at 45, 75, and 105 days after seed emergence (DAS) of each crop. For this purpose, a sample area of one square meter was manually harvested, and the leaf length and maximum width were measured. Then, the leaf area was estimated by multiplying the leaf length and width with the crop-specific co-efficient factor of 0.83 for wheat^61^ and 0.75 for soybean^62^. Afterwards, the LAI of wheat and soybean was measured as the leaf area ratio to the ground area^63^. Additionally, the total LAI of narrow strips, medium strips, and wide strips in WSI was measured from the summation of the wheat LAI and soybean leaf area index.

The dry matter accumulation and distribution analysis of wheat and soybean were measured four times at 45, 75, and 105 DAS and the maturity of each crop. For this purpose, one square meter of wheat and soybean plants were manually harvested. The roots of both crops were collected using the previously published method^64^. After sampling, wheat and soybean plant samples were separated into roots, straw (leaves + stems), and grains and dried in the sun for 10 to 15 days, depending on the weather conditions, to achieve a constant weight. Moreover, the total dry matter of narrow strips, medium strips, and wide strips in WSI was measured from the summation of wheat dry matter and soybean dry matter. Dry matter of wheat or soybean was defined as the sum of all plant parts (roots + straw + grains) at 45, 75, and 105 DAS and maturity. After measuring the dry matter, the same plant samples were used to determine the nitrogen uptake of wheat and soybean under different planting systems at 45, 75, and 105 DAS and maturity. First, the dry plant samples were ground using a hammer mill and then re-ground using a 1 mm screen, and the nitrogen content was measured following the Kjeldahl method^65^. Besides, the total nitrogen uptake of intercropping systems was calculated as the sum of the nitrogen uptake by wheat + and soybean at 45, 75, and 105 DAS and maturity.

At maturity, the grain yield was determined by manually harvesting the 129.6 m^2^ (7.2 m × 18 m) and 259.2 m^2^ (14.4 m × 18 m) area from narrow and medium strips, respectively, while the combine harvester was used to harvest the sample area of 388.8 m^2^ (21.6 m × 18 m), 72 m^2^ (4 m × 18 m), and 72 m^2^ (4 m × 18 m) from wide strips, sole wheat, and sole soybean, respectively. The harvested samples of narrow and medium strips, and threshed samples of wide strips, sole wheat, and sole soybean were then dried in the sun for the next 10–15 days to standard moisture content. Then, the dried wheat and soybean samples were weighed to determine the grain yield of each treatment from every replication. To assess the effect of different planting systems on the yield components of wheat and soybean, approximately one-third of each harvested sample of wheat and soybean were used to estimate the yield components of wheat (spike m^-2^, seeds spike^-1^, hundred seed weight) and soybean (pod plant^-1^, seed plant^-1^, and hundred seed weight) under different intercropping and sole systems. Moreover, the total grain yield of narrow strips, medium strips, and wide strips in WSI was measured from the summation of the wheat grain yield and soybean grain yield^66^.

#### Performance analysis of strip intercropping systems

To calculate the yield advantage of WSI over sole systems, we first measured the partial LER of wheat (pLERw) and soybean (pLERs). The pLERw or pLERs was computed using Eqs. (1) and (2)^67^:

1 $$pLERw=\frac{GYiw}{GRsw}$$

2 $$pLERs=\frac{GYis}{GRss}$$

Where GYis or GYiw is the grain yield of soybean or wheat in WSI, respectively, whereas GYss or GYsw is the grain yield of soybean or wheat in pure stand. Afterwards, the total LER of WSI was estimated using Eq. (3).

3 $$Total LER=pLERw+pLERs$$

To estimate the competition between intercrops in different systems under WSI, we calculated the competition ratio of wheat (CRw) and soybean (CRs). The CRw or CRs was computed using Eqs. (4) and (5)^67^:

4 $$CRw=\frac{pLERw}{pLERs}\times \frac{Zs}{Zw}$$

5 $$CRs=\frac{pLERs}{pLERw}\times \frac{Zw}{Zs}$$

where Zw or Zs represents the sown proportion area of wheat or soybean, respectively, in WSI.

### Economic viability

Economic analysis was done to estimate the economic viability of intercropping soybean into the wheat cropping system and the advantage of the mechanized strip intercropping system (wide strips) over non-mechanized strip intercropping systems (narrow and medium strips). The total expenses for wheat and soybean production in narrow strips, medium strips, wide strips, sole wheat, and sole soybean, including the cost of seeds, fungicides (for seed treatment), fertilizers (urea and diammonium phosphate), pesticides (for borer and sucking insects), and labour, were calculated as per the local market rates. The gross income of all systems was calculated by considering the grain yield based on the local market prices. Then, the net income of each system was estimated as the difference between gross income and total costs. The benefit-to-cost ratio of each system was estimated by dividing gross income by total costs^63,67^.

### Statistical analysis

Significant differences between intercropping (narrow strips, medium strips, and wide strips) and sole cropping (sole wheat and sole soybean) systems were measured using one-way ANOVA with the least significant difference (LSD). The significance was determined at a 5% probability level (*P* < 0.05). Moreover, the figures and tables represent the average values and standard errors of calculated means based on the three replicates per treatment.

## Data Availability

The datasets generated during and/or analyzed during the current study are available from the corresponding author upon reasonable request.
